# Supplementary material for: The relationship between resting-state functional connectivity, antidepressant discontinuation and depression relapse
Source: Sci Rep. 2020 Dec 18;10:22346. doi: 10.1038/s41598-020-79170-9 (PMC7749105; doi:10.1038/s41598-020-79170-9)
Supplement: Supplementary file 1 — Supplementary Information1. [file 41598_2020_79170_MOESM1_ESM.pdf]

Supplementary Material

The relationship between resting-state functional connectivity, antidepressant discontinuation and depression relapse

Isabel M. Berwian[1,2,3*], Julia G. Wenzel[4], Leonie Kuehn[4], Inga Schnuerer[1], Lars Kasper[1,5], Ilya M. Veer[4], Erich Seifritz[2], Klaas E. Stephan[1,6,7], Henrik Walter[4,+] and Quentin J. M. Huys[1,2,8,9,+]

1 Translational Neuromodeling Unit, University of Zurich and ETH Zurich, Zurich, Switzerland **2** Hospital of Psychiatry, University of Zurich, Zurich, Switzerland **3** Princeton Neuroscience Institute, Princeton University Princeton, United States of Zurich **4** Charité Universitätsmedizin, Campus Charité Mitte, Berlin, Germany **5** Institute of Biomedical Engineering, ETH Zurich and University of Zurich, Zurich, Switzerland **6** Wellcome Trust Neuroimaging Centre, University College London, London, United Kingdom **7** Max Planck Institute for Metabolism Research, Cologne, Germany **8** Division of Psychiatry and Max Planck Centre for Computational Psychiatry and Ageing Research, University College London, London, United Kingdom **9** Camden and Islington NHS Foundation Trust, London, United Kingdom **+** Equal contribution * **Corresponding author**: Isabel Berwian, iberwian@princeton.edu

S1 Supplementary Methods

S1.1 In- and Exclusion Criteria

Participants fulfilling the following inclusion criteria were eligible for participation in the study:

1. age 18-55 years
2. ability to consent and adhere to the study protocol
3. written informed consent
4. fluent in written and spoken German.

Patients had to additionally fulfil the following criteria:

1. currently under medical care with a psychiatrist or general practitioner for remitted Major Depressive Disorder and willing to remain in care for the duration of the study (approx. 9 months)
2. informed choice to discontinue medication (including willingness to taper the medication over at most 12 weeks) that was independent of study participation
3. clinical remission (Hamilton Depression Score of less than 7) had been achieved under therapy with Antidepressant Medication (ADM) without having undergone manualized psychotherapy; with no other concurrent psychotropic medication and had been maintained for a minimum of 30 days,
4. consent to information exchange between treating physician and study team members regarding inclusion/exclusion criteria and past medical history.

Any of the following exclusion criteria led to exclusion of an participant. This included the following general criteria

1. any disease of type and severity sufficient to influence the planned measurement or to interfere with the parameters of interest (This includes neurological, endocrinological, oncological comorbidities, a history of traumatic or other brain injury, neurosurgery or longer loss of consciousness.)
2. premenstrual syndrome (ICD-10 N94.3).

and MRI-related criteria

1. MRI-incompatible metal parts in the body,
2. inability to sit or lie still for a longer period,
3. possibility of presence of any metal fragments in the body,
4. pregnancy,
5. pacemaker, neurostimulator or any other head or heart implants,
6. claustrophobia and
7. dependence on hearing aid.

For patients the following additional criteria would led to exclusion:

1. current psychotropic medication other than antidepressants,
2. questionable history of major depressive episodes without complicating factors,
3. current acute suicidality,
4. lifetime or current axis II diagnosis of borderline or antisocial personality disorder,

5. lifetime or current psychotic disorder of any kind, bipolar disorder,
6. current posttraumatic stress disorder, obsessive compulsive disorder, or eating disorder
7. current drug use disorder (with the exception of nicotine) or within the past 5 years.

Healthy controls were excluded if there was a lifetime history of Diagnostic and Statistical Manual of Mental Disorders (4th ed., text rev.)(1) axis I or axis II disorder with the exception of nicotine dependence.

S1.2 Questionnaires and Clinical Assessments

Clinical in- and exclusion criteria were assessed with the Structured Clinical Interview for DSM-IV (SCID) I and II to diagnose axis 1 disorders (major mental disorders) and axis II disorders (personality disorders), respectively(2). The Structured Interview Guide for Hamilton Depression Rating Scale (SIGH-D)(3) consisting of 17 items was used to assess inclusion and the Inventory of Depressive Symptomatology Clinician Rated (IDS-C)(4) with 30 items to quantify residual depression. Additionally, we applied the German version of the Response Style Questionnaire (RSQ-10D)(5) measuring brooding and reflection as components of rumination with 5 items each.

S1.3 Data Analysis

All analyses, except for the preprocessing of the imaging data, were performed using Matlab version 2016b.

We computed an overall measure of disease severity as the first principal component of number of past depressive episodes, age at illness onset, time in remission, time since depression onset, severity of last episode, time sick in total and time sick in the last five years as variables.

Medication load was based on the dose prior to discontinuation divided by the maximal allowed dose according to the Swiss compendium (www.compendium.ch) and by the weight of the participant.

Psychotherapy score was coded such that patients with no psychotherapy within the year before the study received a 0, patients reporting to have completed a psychotherapy within one year before the study a 0.5 and patients reporting to be in psychotherapy at the beginning of the study as 1. Significance was computed with a three-way chi-squared test.

S1.4 Image Acquisition

Images were acquired at the two study sites using a Phillips 3T Ingenia in Zurich and a Siemens 3T Trio in Berlin. Participants were instructed to stay awake, keep their eyes open and look at a centrally placed fixation cross.

In Zurich, a 32-channel coil was used to acquire echo-planar images (EPIs; 136 volumes; 40 axial slices; 2.5mm slice sickness; descending sequential acquisition, repetition time: 2560 ms; echo time: 27, field of view: 210 x 210 x 119.5, acquisition matrix: 84 x 82, reconstructed voxel size: 2.19 x 2.19 x 2.50 mm, flip angle: 90°). Additionally, we acquired T1-weighted magnetization-prepared rapid-acquisition gradient-echo (MPRAGE) structural images (301 axial slices; slice thickness: 1; repetition time: 7.9ms; echo time: 3.7ms, field of view: 250 x 250 x 180.6, acquisition matrix: 252 x 251, reconstructed voxel size: 0.98 x 0.98 x 0.60 mm, flip angle: 8°).

In Berlin, a 32-channel coil was used for functional resting-state EPIs (136 volumes; 40 axial slices; 3mm slice thickness including a gap of 0.5mm; descending sequential acquisition, repetition time: 2560 ms; echo time: 27

ms, field of view: 210 x 210 x 120, acquisition matrix: 84 x 84, voxel size: 2.50 x 2.50 x 2.50 mm, flip angle: 90°). T1-weighted MPRAGE structural images (192 axial slices; slice thickness: 1mm; repetition time: 1900 ms; echo time: 2.52 ms, field of view: 256 x 256 x 192, acquisition matrix: 256 x 256, reconstructed voxel size: 0.98 x 0.98 x 0.60mm, flip angle: 9°) were also acquired.

S1.5 Preprocessing

Functional images were realigned, slice-time corrected and smoothed with a 6mm FWHM kernel using adaptive spatial procedure (SUSAN(6)) in FSL (FMRIB Software Library v5.0). The images were then co-registered to the structural image and normalised using Advanced Normalization Tools (ANTs(7)). Finally, an independent component analysis-based artefact removal (ICA-AROMA(8)) was applied to exclude noise components relating e.g. to breathing and heart rate, using a data-driven approach and the data was subjected to a high-pass filter of 0.008Hz. Lastly, BOLD data were normalised to MNI standard space, applying the registration matrices and warp images from the two previous registration steps, and then resampled into 2 mm isotropic voxels. All imaging data were visually inspected to exclude acquisition artefacts or other data corruption.

S1.6 Motion correction

As group differences can be confounded by head motion differences(11), we excluded participants from all analyses if their frame-wise displacement (FD) from one volume to the next exceeded 1mm at any time during the scan. To test for the effects of motion, we performed a median-split based on the mean FD and compared RSFC for all seeds between all participants included at MA1. In case effects were negligible, we used 6 realignment parameters as motion regressors on the first level and no further correction to avoid over-fitting and power reduction. In case non-negligible motion artefacts were observed, we would have additionally added the 6 derivatives of the realignment parameters and censored those scans for which FDs were bigger than 0.5. Censoring scans means to include an additional regressor for each volume at which the movement exceeds a given threshold, here 0.5 FD. This regressors contains zeros at all volumes but the volume that exceeds the threshold. At that volume, the regressor contains a one.

S1.7 Study site effects

To examine systematic differences between the two study sites, we compared the temporal signal-to-noise ratio in the grey matter for all included subjects between sites.

S1.8 Affective mask creation

The "affective mask" consists of functional and anatomical masks that were merged in SPM.

The following regions of interest (ROIs) were taken from the CONN toolbox(9) to build masks for the default mode network, the salience network and the executive (dorsal attention and fronto parietal) network defined by ICA analyses of 497 subjects from the human connectome project in the toolbox.

DefaultMode.MPFC (1,55,-3)
DefaultMode.LP (L) (-39,-77,33)

DefaultMode.LP (R) (47,-67,29)
DefaultMode.PCC (1,-61,38)
Salience.ACC (0,22,35)
Salience.AInsula (L) (-44,13,1)
Salience.AInsula (R) (47,14,0)
Salience.RPFC (L) (-32,45,27)
Salience.RPFC (R) (32,46,27)
Salience.SMG (L) (-60,-39,31)
Salience.SMG (R) (62,-35,32)
DorsalAttention.FEF (L) (-27,-9,64)
DorsalAttention.FEF (R) (30,-6,64)
DorsalAttention.IPS (L) (-39,-43,52)
DorsalAttention.IPS (R) (39,-42,54)
FrontoParietal.LPFC (L) (-43,33,28)
FrontoParietal.PPC (L) (-46,-58,49)
FrontoParietal.LPFC (R) (41,38,30)
FrontoParietal.PPC (R) (52,-52,45)

Amygdala and hippocampus masks:
These masks were built using the SPM Anatomy toolbox(10). We used anatomical ROIs to create the right and left amygdala labelled AStr, CM, LB and SF in the toolbox. Similarly, we used anatomical ROIs to create the right and left hippocampus labelled CA1, CA2, CA3 and DG in the toolbox.

S1.9 Sanity checks and exploratory analyses

To specifically examine effects of time, paired t-test in patients who did not discontinue but were assessed twice (group MA1-MA2-D) were conducted.

To ensure the validity of our method, we repeated the analyses without adding the covariates from aCompCor in the first level. We also repeated the analyses without adding motion regressors at that stage.

To explore whether we missed strong abnormalities that were outside our restricted search volume, i.e. the affective mask, which might be of interest for future studies, we repeated all second level analyses without the affective mask in whole-brain analyses. In addition, we report results without correction for multiple comparison for number of seeds and uncorrected results at a significance level of 0.001 for all main seed analyses to allow for estimates of potential type II errors.

S2 Supplementary Results

S2.1 Quality checks

To ensure that functional ectivity between our chosen seeds and the anticipated networks based on the literature was evident, we visually inspected the networks connected to the seeds in all participants included for analyses

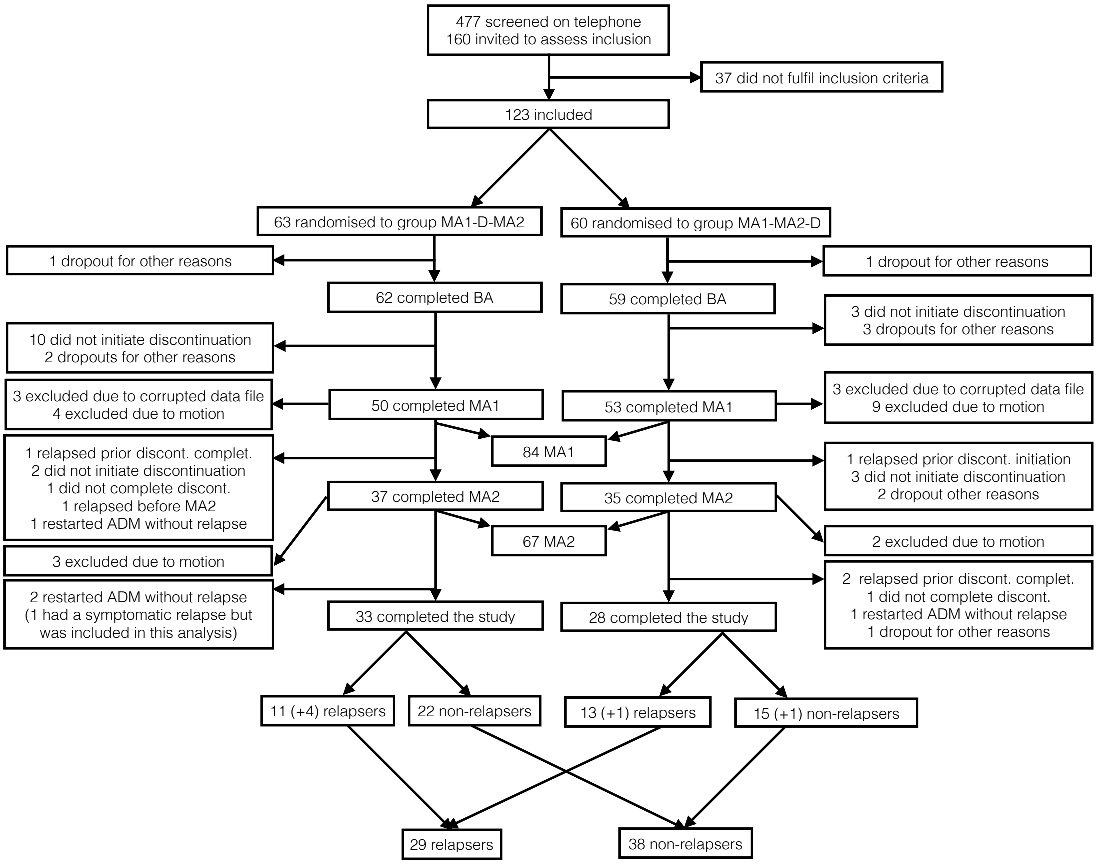

Figure S1: Consort Diagram for Patients: Depicted are reasons for dropouts and exclusion for patients throughout the study. (+ X) indicates the number of participants who either relapsed or did not relapse but did not have useable data at main assessment (MA) 2. MA1-D-MA2 = Discontinuation between MA1 and MA2; MA1-MA2-D = MA1 and MA2 before Discontinuation; BA = baseline assessment

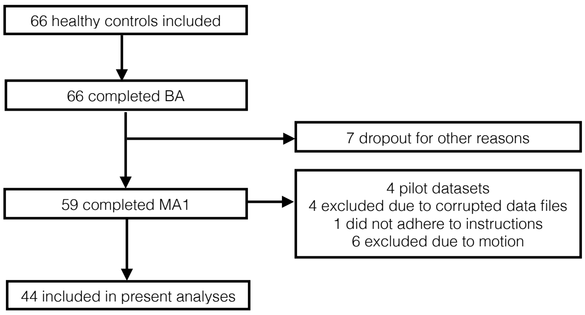

Figure S2: Consort Diagram for Healthy Controls: Depicted are reasons for dropouts and exclusion for controls throughout the study. BA = baseline assessment; MA = main assessment

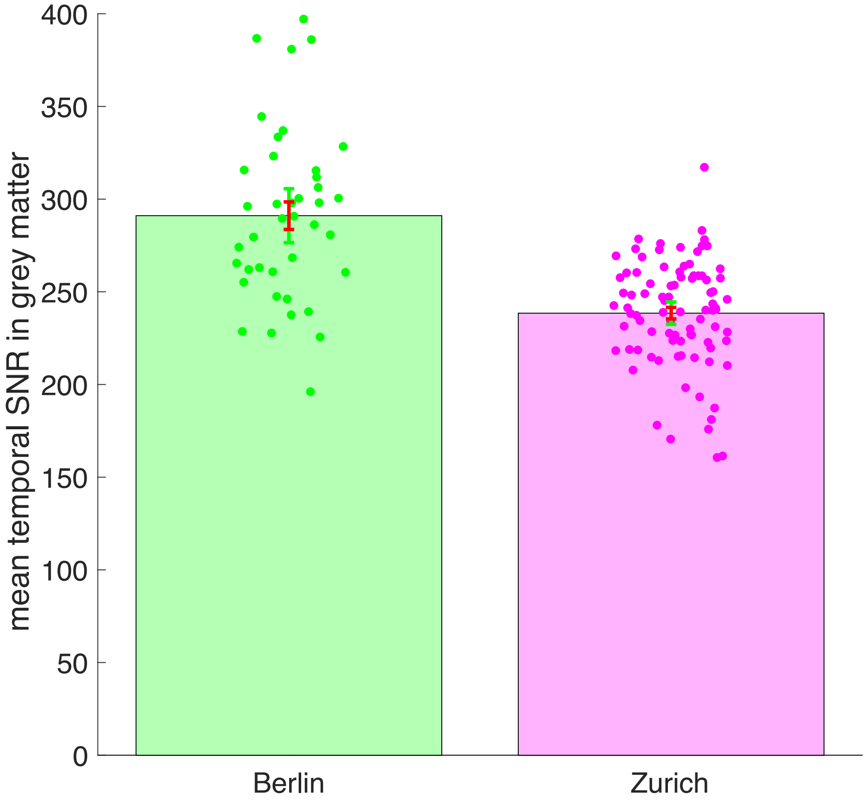

Figure S3: Site effects: Depicted is the average signal-to-noise ratio (SNR) within the individual grey matter masks over the time of the resting-state period. Dots indicate individual data points, red error bars show standard errors and green error bars show 95% confidence intervals.

at MA1. Figure S4 depicts these networks for all seeds in the left hemisphere. Network functional connectivity seems as expected.

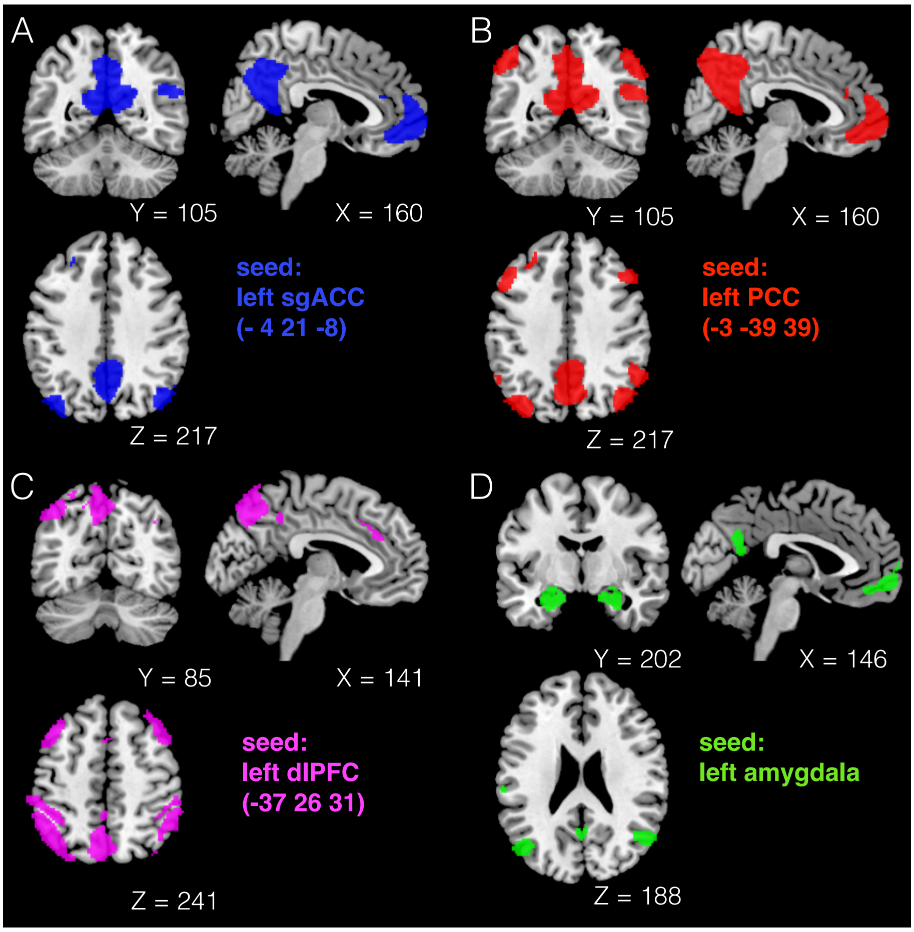

Figure S4: Functional connectivity networks of all left-sided seeds: sgACC = subgenual anterior cingulate cortex; PCC = posterior cingulate cortex, dlPFC = dorsolateral prefrontal cortex

S2.2 Effects of time

There were no significant changes in RSFC for any of the seeds in patients who were assessed twice prior to discontinuation.

S2.3 Effects of noise regressors on the first level

Analyses without regressors for motion on the first level replicated the main pattern of results. Not including additional regressors from aCompCor in the first level analyses also replicated the main pattern of results.

S2.4 Whole-brain exploratory analyses

Repeating all second level analyses without the affective mask led to the similar significant clusters as reported for the within mask analyses, whereas the p-values naturally differed (parietal cortex: p=0.021, PCC: p=0.004). Of note, no additional effects emerged.

S2.5 Uncorrected results

Table S1 depicts results for all main seeds considered significant at 0.001 without correction. The sparsity of results at this significance level speaks against a high rate of type II error due to correction for multiple comparison, but supports the null hypotheses for many of the examined effects.

Table S1: Significant results at uncorrected level for main seeds

Ssed	Contrast	BA/Region	Peak MNI coordinates			k	p	T-Value	Z	p
		x	y	z			(uncor.)			(FWE-cor., peak)
**left sgACC**	T-test for Pat - HC	Left BA 10	-34	48	14	1	0.001	3.26	3.19	0.821
	T-test for HC - Pat	Left BA 8	-34	22	58	1	0.001	3.30	3.22	0.795
	T-test for No Rel - Rel	Right BA 19	48	-72	26	11	< 0.001	3.60	3.45	0.612
	T-test for No Rel - Rel	Right sensory assoc.	28	-42	64	1	0.001	3.25	3.11	0.942
**left PCC**	F-test discontinution	Left BA 7	-38	-48	56	26	< 0.001	4.35	4.19	0.059
	F-test discontinution	Right amygdala	16	-2	-18	8	< 0.001	3.56	3.47	0.522
	F-test discontinution	Right amygdala	24	4	-26	2	< 0.001	3.53	3.44	0.556
	F-test discont. x rel.	Right BA 10	40	58	-8	3	< 0.001	3.78	3.57	0.432

T-tests are independent sample t-tests. F-tests show interactions for the discontinuation effect and the discontinuation x relapse interaction effect. BA = Brodmann area, MNI = Montreal Neurological Institue, sgACC = subgenual anterior cingulate cortex, HC = healthy controls, Pat. = patients, No Rel = no relapse, Rel = relapse, PCC = posterior cingulate cortex

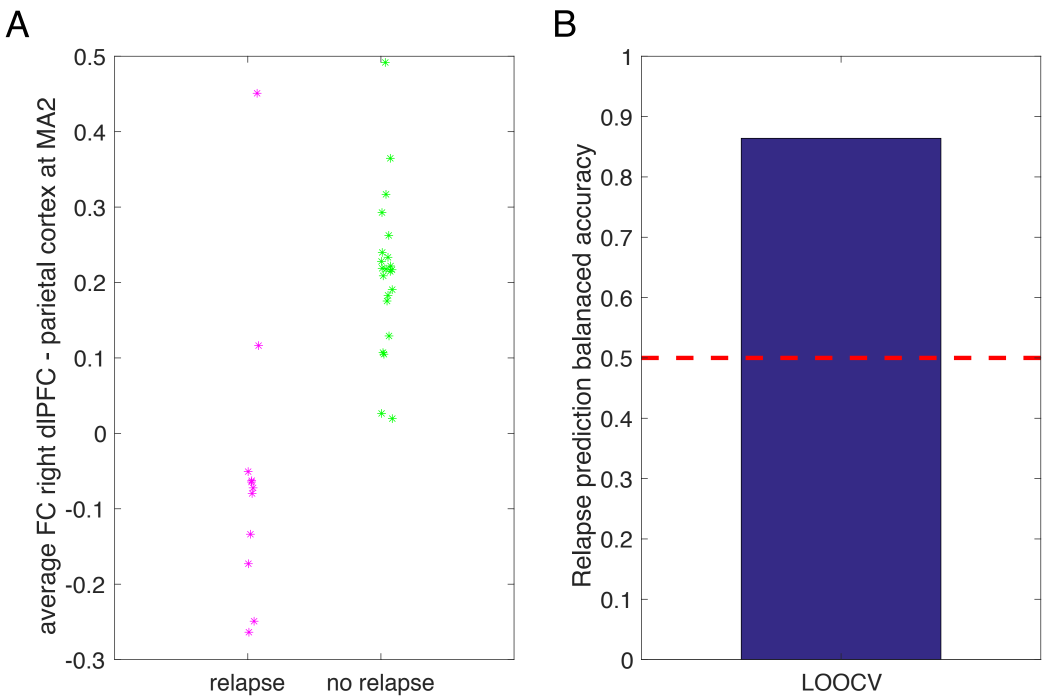

Figure S5: Prediction analyses: A) Average functional connectivity (FC) for the significant cluster in the parietal cortex at main assessment 2 (MA2) B) Balanced accuracy for predicting subsequent relapse using leave-one-out cross-validation. The dashed red line indicates chance level. dlPFC = dorsolateral prefrontal cortex

References

[1] American Psychiatric Association. *Diagnostic and Statistical Manual of Mental Disorders 4th ed., text rev.* (Author, Washington, DC, 2000).

[2] Wittchen, H.-U. & Fydrich, T. *Strukturiertes klinisches Interview für DSM-IV. Manual zum SKID-I und SKID-II* (Hofgrefe, Göttingen, DE, 1997).

[3] Williams, J. B. A structured interview guide for the hamilton depression rating scale. *Arch Gen Psychiatry* **45**, 742–7 (1988).

[4] Rush, A. J., Gullion, C. M., Basco, M. R., Jarrett, R. B. & Trivedi, M. H. The inventory of depressive symptomatology (IDS): psychometric properties. *Psychol Med* **26**, 477–86 (1996).

[5] Huffziger, S. & Kühner, C. Die Ruminationsfacetten Brooding und Reflection: Eine psychometrische Evaluation der deutschsprachigen Version RSQ-10D. *Z Klin Psychol und Psychother* **41**, 38–46 (2012).

[6] Smith, S. M. & Brady, J. M. SUSAN - A new approach to low level image processing. *Int. J. Comput. Vis.* **23**, 45–78 (1997).

[7] Avants, B. B. *et al.* A reproducible evaluation of ants similarity metric performance in brain image registration. *Neuroimage* **54**, 2033–44 (2011).

[8] Pruim, R. H. R. *et al.* ICA-AROMA: A robust ICA-based strategy for removing motion artifacts from fMRI data. *Neuroimage* **112**, 267–277 (2015).

[9] Whitfield-Gabrieli, S. & Nieto-Castanon, A. Conn: a functional connectivity toolbox for correlated and anticorrelated brain networks. *Brain Connect* **2**, 125–41 (2012).

[10] Eickhoff, S. B. *et al.* A new spm toolbox for combining probabilistic cytoarchitectonic maps and functional imaging data. *Neuroimage* **25**, 1325–35 (2005).

[11] Power, J. D., Barnes, K. A., Snyder, A. Z., Schlaggar, B. L. & Petersen, S. E. Spurious but systematic correlations in functional connectivity MRI networks arise from subject motion. *Neuroimage* **59**, 2142–54 (2012).
